# CAR T Cell-Based Immunotherapy for the Treatment of Glioblastoma

**DOI:** 10.3389/fnins.2021.662064

**Published:** 2021-05-25

**Authors:** Luke Maggs, Giulia Cattaneo, Ali Emre Dal, Ali Sanjari Moghaddam, Soldano Ferrone

**Affiliations:** Department of Surgery, Massachusetts General Hospital and Harvard Medical School, Boston, MA, United States

**Keywords:** CAR T cell, clinical trial, glioblastoma, immunotherapy, preclinical, tumor antigen

## Abstract

Glioblastoma multiforme (GBM) is the most common and aggressive malignant primary brain tumor in adults. Current treatment options typically consist of surgery followed by chemotherapy or more frequently radiotherapy, however, median patient survival remains at just over 1 year. Therefore, the need for novel curative therapies for GBM is vital. Characterization of GBM cells has contributed to identify several molecules as targets for immunotherapy-based treatments such as EGFR/EGFRvIII, IL13Rα2, B7-H3, and CSPG4. Cytotoxic T lymphocytes collected from a patient can be genetically modified to express a chimeric antigen receptor (CAR) specific for an identified tumor antigen (TA). These CAR T cells can then be re-administered to the patient to identify and eliminate cancer cells. The impressive clinical responses to TA-specific CAR T cell-based therapies in patients with hematological malignancies have generated a lot of interest in the application of this strategy with solid tumors including GBM. Several clinical trials are evaluating TA-specific CAR T cells to treat GBM. Unfortunately, the efficacy of CAR T cells against solid tumors has been limited due to several factors. These include the immunosuppressive tumor microenvironment, inadequate trafficking and infiltration of CAR T cells and their lack of persistence and activity. In particular, GBM has specific limitations to overcome including acquired resistance to therapy, limited diffusion across the blood brain barrier and risks of central nervous system toxicity. Here we review current CAR T cell-based approaches for the treatment of GBM and summarize the mechanisms being explored in pre-clinical, as well as clinical studies to improve their anti-tumor activity.

## Introduction

Gliomas are the most common type of primary brain cancer (Weller et al., 2015; Ostrom et al., 2019). They originate from brain cells including astrocytes, oligodendrocytes, and ependymal cells, which support neural cells. Gliomas can be divided into six types based on their histological characteristics; among them glioblastoma multiforme (GBM), classified as a grade IV glioma, is the most common malignant primary brain tumor. GBM, which originates from astrocytes, the most abundant cell type in the central nervous system (CNS), is highly aggressive and has an extremely unfavorable prognosis. Current treatment options typically consist of surgery followed by chemotherapy or radiotherapy with a dismal 2 years patient survival rate of less than 30% (Ostrom et al., 2019). While several studies are being performed in attempts to optimize combinatorial strategies incorporating radiotherapy and/or chemotherapy, the disappointing clinical results generated by these therapies emphasize the urgent need to develop novel and more effective treatments.

Several immunotherapeutic strategies have been tested in clinical trials in patients with GBM; surprisingly the impact on clinical outcomes has been limited. Recently there has been a growing interest in the application of immunotherapy which utilizes as an effector mechanism a patient’s own T cells transduced with a tumor antigen (TA)-specific chimeric antigen receptor (CAR). This approach has generated impressive clinical responses in patients with hematological malignancies (Boyiadzis et al., 2018), but has had thus far limited, if any success in patients with solid tumors, including GBM. The latter results most likely reflect the limited specificity of the target TA, the immune escape mechanisms facilitated by the hostile conditions of the tumor microenvironment (TME) and the limited ability of CAR T cells to infiltrate the tumor site as well as their insufficient activity and persistence. This information is being used for the rational design of strategies to enhance the efficacy of CAR T cell-based immunotherapy of patients with solid cancers. The exciting potential of these strategies is indicated by the large number of investigators who are testing them both in preclinical models and in clinical trials.

**FIGURE 1:**
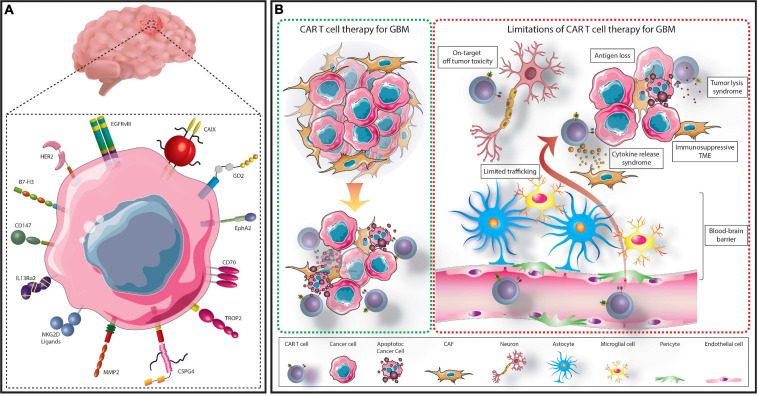
Summary of CAR T cell treatment for GBM. GBM cells express several targetable TAs which have either been assessed in clinical or preclinical CAR T cell studies **(A)**. TA-specific CAR T cells can be administered to patients to recognize and eliminate TA expressing GBM cells **(B)**. However, several limitations hinder this process. These include; the immunosuppressive TME, limited access across the BBB, on-target, off-tumor toxicity, cytokine release syndrome, tumor lysis syndrome, and selective antigen loss.

In this review, we will briefly discuss the current range of immunotherapeutic strategies being explored to treat GBM before focusing specifically on CAR T cell therapy. TA being utilized as targets in CAR T cell based preclinical and clinical studies will be reviewed followed by a discussion of the variables which limit CAR T cell therapy against GBM (Figure 1). Finally, we will describe the novel strategies and combinatorial therapies which are being tested in pre-clinical studies to enhance the antitumor activity of CAR T cells with the expectation that they may overcome these limitations.

## Immunotherapy Based Strategies for the Treatment of GBM

Current immunotherapeutic strategies for the treatment of GBM are mainly focused on cognate T cell-based therapies, such as the use of vaccines and immune checkpoint inhibitors (ICI), or on TA-specific monoclonal antibodies (mAb), either alone or conjugated to an antitumor drug, toxin or radioisotope (Gan et al., 2017; Choi et al., 2019a; Medikonda et al., 2020). Each strategy has advantages and limitations, which in combination with the specific challenges imposed by GBM, have thus far failed to provide significant improvements in patient outcomes (Table 1).

**TABLE 1:** Immunotherapeutic strategies tested in patients with GBM.

Immunotherapy	Strengths	Weaknesses	Applied to GBM patients	References
Antibodies	High specificity and affinity Low cost	Low efficacy Limited ability to cross BBB Off target toxicity	Bevacizumab (anti-VEGF) Nimotuzumab (anti-EGFR) Cetuximab (anti-EGFR)	Neyns et al., 2009; Westphal et al., 2015; Lombardi et al., 2017
Adoptive effector cell transfer	Ex vivo stimulation and expansion of tumor cell targeting immune cells Safe (auto)	Poor survival and proliferation Poor trafficking to tumor sites Risk of GvHD (allo)	Allogeneic T cells Autologous T cells Autologous lymphokine-activated killer cells Autologous tumor infiltrating lymphocytes	Quattrocchi et al., 1999; Weenink et al., 2020
CAR T cells	Specificity HLA independent Long term proliferation and survival (‘Living drug’)	Costly and time-consuming manufacture Potential toxicity Acquired resistance	EGFRvIII-specific CAR T cells HER2-specific CAR T cells IL13Rα2-specific CAR T cells	Brown et al., 2015; Ahmed et al., 2017; Goff et al., 2019
Checkpoint Inhibitors	Easily applicable Proven response in certain subsets of patients	Reliant on cognate T cells Reliant on HLA class I APM function in cancer cells Variable response Lack of markers to identify potentially responding patients	Pembrolizumab (anti-PD-1) Nivolumab (anti-PD-1) Nivolumab and ipilimumab (anti-CTLA-4) Durvalumab (anti-PD-L1) Avelumab (anti-PD-L1)	Cloughesy et al., 2019; Schalper et al., 2019; Reardon et al., 2020
Oncolytic Viruses	Specificity Low toxicity	Low anti-tumor efficacy Dependent on enhancement of host immune system response Dependent on cancer cell susceptibility to the induced effector mechanism	HSV-1 M032 DNX-2401 AdV-tK Reolysin G207	Markert et al., 2000; Forsyth et al., 2008; Patel et al., 2016; Kieran et al., 2019; Philbrick and Adamson, 2019
Vaccines	Specificity Safe Patient specific Ability to target multiple TAs	Variable immune response Reliant on cognate T cell efficacy (HLA restricted) Identification of immunogenic source required Can be costly	Peptide vaccines: Autologous peptides EGFRvIII protein Multiple tumor antigens Survivin Dendritic cell vaccines: Tumor lysate Glioma stem like cell antigens	Weller et al., 2017; Liau et al., 2018; Weenink et al., 2020

### T Cell-Based Immunotherapeutic Strategies: Vaccines

Cancer vaccines are constructed using isolated cancer peptides, synthesized peptides derived from TA amino acid sequences or dendritic cells (DCs) pulsed with purified cancer TAs or an autologous or allogeneic tumor cell lysate. Several ongoing vaccine-based clinical trials are in early Phase I/II trials. Two therapeutic vaccines reached the Phase III stage of clinical testing in newly diagnosed GBM patients: one utilized autologous tumor cell lysate-pulsed dendritic cell vaccine (DCVax) (NCT00045968) (Liau et al., 2018), and the other one utilized Rindopepimut, an EGFR variant III (EGFRvIII)-specific peptide conjugated to keyhole limpet haemocyanin (NCT01480479) (Weller et al., 2017; Lim et al., 2018). Unfortunately, both displayed minimal clinical benefit. These disappointing clinical results are likely to reflect the many limitations of T cell-based therapeutic strategies based on vaccination. They include: (i) the extent of immune response is generally insufficient to achieve clinical benefits, (ii) the immune response to vaccination is highly variable among immunized patients, (iii) the defective TA derived peptide presentation to cognate T cells caused by abnormalities in HLA class I antigen processing machinery (APM) component expression and/or function frequently present in GBM cells provides them with an escape mechanism from immune surveillance and from the TA-specific immune response mounted by the immunized host and, (iv) the costly and time-consuming preparation of cancer vaccines imposes strictures on their production (Oh et al., 2015; Lim et al., 2018).

### T Cell-Based Immunotherapeutic Strategies: Immune Checkpoint Inhibitors (ICI)

Immune checkpoint specific mAbs have been used to unleash T cells which recognize cancer cells through blockade of inhibitory signaling pathways (Abril-Rodriguez and Ribas, 2017). They have induced impressive clinical responses in several cancer types (Hodi et al., 2010; Ansell et al., 2015; Borghaei et al., 2015). ICIs targeting cytotoxic T-lymphocyte associated protein 4 (CTLA-4), programmed cell death 1 (PD-1) or indoleamine 2,3-dioxygenase 1 (IDO) have yielded encouraging preclinical results in mouse models of glioma (Zeng et al., 2013; Wainwright et al., 2014). However, they have failed to demonstrate survival benefit for GBM patients in a clinical setting. The first large-scale clinical trial designed to evaluate the safety and efficacy of the anti-PD-1 Nivolumab in patients with recurrent GBM (CheckMate-143: NCT02017717) did not prolong patients’ overall survival (OS) (Filley et al., 2017; Mantica et al., 2018).

Factors limiting the efficacy of T cell-based therapeutic strategies include the low mutational burden of GBM, the limited trafficking of immune cells within the tumor and the highly suppressive nature of the tumor microenvironment (TME) (Desai et al., 2019). Another important mechanism that plays a role in the limited clinical impact of T cell-based immunotherapies is represented by defects in HLA class I and APM component expression and function in GBM (Facoetti et al., 2005; Thuring et al., 2015). Downregulation, loss or lack of function of HLA class-I APM components have a negative impact on T cell-mediated recognition and lysis of GBM cells.

### Oncolytic Virus-Based Immunotherapy

Several viruses designed to directly kill cancer cells via oncolysis and to stimulate an immune response to the released TAs have been tested in many types of solid cancer (Lawler et al., 2017) including GBM (Iorgulescu et al., 2018; Martikainen and Essand, 2019). Phase I/II clinical trials have shown the feasibility and safety of this approach but have yielded limited clinical benefit in the majority of patients (Cloughesy et al., 2018; Desjardins et al., 2018; Lang et al., 2018). In some GBM patients oncolytic viruses can significantly improve survival (Gesundheit et al., 2020). However, further investigation into the molecular mechanisms involved are needed to improve this therapeutic strategy. Attempts to enhance the effect of oncolytic viruses through addition of a therapeutic payload such as a cytokine, chemotherapeutic agent or ICI are also under investigation (Xu B. et al., 2018; Martikainen and Essand, 2019; Passaro et al., 2019).

### Antibody Based Immunotherapies: mAbs Targeting Molecules Crucial for Cell Survival or TAs

The anti-VEGF mAb Bevacizumab has been used to treat patients with GBM for over a decade with limited survival advantage in the recurrent setting (Diaz et al., 2017; Wick et al., 2017). MAbs targeting EGFRvIII have been administered to patients with newly diagnosed GBM, however, they were unable to significantly inhibit the signaling pathway and improve survival (Westphal et al., 2015). Likewise mAbs targeting EGFR signaling linked to toxins such as *Pseudomonas aeruginosa* exotoxin A (PE) and diphtheria toxin (DT) or to radioisotopes including Iodine-125 and Rhenium-188 as cytotoxic payloads have also yielded relatively disappointing results in patients (Gan et al., 2017). Antibody drug conjugates (ADCs) such as ABT-414 (an anti-EGFR mAb conjugated to tubulin assembly inhibitor monomethyl auristatin F) and AMG 595 (an anti-EGFRvIII mAb conjugated to microtubule-assembly inhibitor maytansinoid DM1) have been tested in clinical trials (NCT01800695; NCT01475006) (Hamblett et al., 2015; Lassman et al., 2015). Results with these agents have been more promising and have encouraged targeting of alternative antigens expressed on GBM cells. An ongoing Phase I/II clinical trial (NCT01631552) is targeting TROP2 in patients with epithelial cancers or other types of cancer, including GBM; this clinical trial utilizes an ADC (IMMU-132) which contains SN-38, the active metabolite of the chemotherapeutic agent irinotecan, linked to the anti-TROP2 antibody hRS7. This ADC construct has shown encouraging results in the treatment of metastatic breast cancer (Zaman et al., 2019). Furthermore, preclinical data obtained both *in vitro* and *in vivo* have proven the efficacy of ADC and radiolabeled antibody approaches targeting Ephrin type-A receptor 3 (EphA3) expressed by GBM cells (Offenhäuser et al., 2018). Similarly the use of an anti-integrin α10 antibody conjugated to the cytotoxin saporin (anti-α10-SAP) exerted antitumor effects against GBM cell lines both *in vitro* and in an orthotopic xenograft mouse model of GBM (Munksgaard Thorén et al., 2019). These results altogether indicate the potential of ADC antitumor activity for the treatment of GBM. However, low efficacy, difficulties with drug delivery, toxicity, acquired resistance and inadequate antitumor activity *in vivo* have markedly reduced the enthusiasm for this type of antibody based therapy (Gan et al., 2017), which in general no longer appears to be a primary interest within the field of immunotherapy of hematological and solid malignancies.

### CAR T Cell Therapy to Treat GBM

The development of cellular engineering technology has resulted in the ability to genetically modify T cells to express a TA-specific CAR. Impressive clinical responses produced by CD19-specific CAR T cells in the field of hematological malignancies (Porter et al., 2015; Schuster et al., 2017; Boyiadzis et al., 2018) have led to FDA approval of three CAR T cell-based therapies (Beyar-Katz and Gill, 2020; Holstein and Lunning, 2020). These remarkable clinical responses have stimulated interest in developing and applying CAR T cell based therapeutic strategies for the treatment of solid tumors including GBM (Choi et al., 2019a).

Chimeric antigen receptors are recombinant receptors typically composed of an extracellular antigen-recognition moiety, mostly (although not exclusively) derived from an antibody that is linked, via spacer/hinge and transmembrane domains, to intracellular signaling domains. The latter include costimulatory domains and T-cell activation moieties, which have been optimized in successive generations of CAR T cells to enhance the signaling activity. T cells grafted with CARs acquire the ability to specifically recognize cancer cells and lyse them. As the recognition and effector mechanism of CAR T cells is HLA class I independent, this type of therapy is not negatively impacted by the downregulation or defective presentation of TAs due to structural and/or functional abnormalities in HLA class I APM components, which often occur in GBM (Facoetti et al., 2005; Thuring et al., 2015). It is noteworthy that T cell receptors can only recognize short peptide sequences. In contrast CARs have the flexibility to be able to recognize TAs in several forms such as carbohydrates, glycolipids and proteins (Abbott et al., 2020) which do not have to be processed for their recognition. However, a limitation of CARs is that with rare exceptions (Maus et al., 2012) they require that the target TA is expressed on the cell membrane, while T cell receptors recognize mostly intracellular moieties which are transported to the cell membrane by MHC class I antigens.

Another major limitation in the antitumor activity of CAR T cell-based immunotherapy is the selectivity and heterogeneity of the targeted TA expression. An ideal target is expected to be homogenously expressed on all differentiated and cancer initiating cells (CICs) within a primary tumor and in metastases. CICs is a practical term used to indicate cells which are in an early stage of differentiation within a lineage, including cancer stem cells (Zhou et al., 2009). These cells are resistant to conventional chemo- and radio-therapy (Maccalli et al., 2018; Steinbichler et al., 2018). Growing experimental evidence argues in favor of the possibility that elimination of CICs is a crucial requirement for a therapy to be curative because these cells appear to play a major role in disease recurrence and metastatic spread. In addition, the TA should be undetectable or have such a minimal expression on normal tissues that it will not mediate elimination of normal cells by the CAR T cells which recognize it. Toxicity and cytokine release syndrome caused by the targeting of normal cells are potentially significant side effects of CAR T cell therapy and highlight the need for Phase I trials to test their safety and feasibility.

Immunohistochemical (IHC) analysis of GBM tumors has helped identify several molecules as potential targets for CAR T cell-based immunotherapy treatments; many of them have been used in clinical trials (Tables 2, 3). In the next section we will discuss the target TAs currently in clinical and preclinical studies. We will describe first the CARs which have been tested in clinical trials, dividing them into those for which clinical trials have been completed and those for which clinical trials are on-going. Then we will describe the CARs which are being tested in preclinical models of GBM.

**TABLE 2:** Completed CAR T cell-based clinical trials in patients with GBM.

Molecular target	Clinical trial identifier and title	Study phase	CAR T cell dosage (+ combination)	Sponsor/site (+ collaborators)	Enrolment	Response
**EGFRvIII**	**NCT02209376** Autologous T Cells Redirected to EGFRVIII-With a Chimeric Antigen Receptor in Patients With EGFRVIII+ Glioblastoma	1	Intravenous single dose of 1.75 × 10^8^–5 × 10^8^ CAR T cells	University of Pennsylvania (University of California)	11	**Median overall survival** ∼8 months, nil benefit Terminated (to pursue combination therapies) (O’Rourke et al., 2017)
	**NCT01454596** CAR T Cell Receptor Immunotherapy Targeting EGFRvIII for Patients With Malignant Gliomas Expressing EGFRvIII	1/2	Two intravenous doses of 6.3 × 10^6^ to 2.6 × 10^10^ CAR T cells per infusion, 2 h apart	National Cancer Institute	18	**Median overall survival** 6.9 months **Median Progression-free survival** 1.3 months, nil benefit (Goff et al., 2019)
**HER2**	**NCT01109095** CMV-specific Cytotoxic T Lymphocytes Expressing CAR Targeting HER2 in Patients With GBM (HERT-GBM)	1	One or more intravenous infusion of 1 x 10^6^/m^2^ – 1 x 10^8^/m^2^ CAR T cells	Baylor College of Medicine (The Methodist Hospital System, Center for Cell and Gene Therapy)	16	**Median overall survival** 24.5 months **Median progression-free survival** 3.5 months, 1 (6%) patient had partial response, 7 (44%) had a stable disease (Ahmed et al., 2017)
**IL13Rα2**	**NCT00730613** Cellular Adoptive Immunotherapy Using Genetically Modified T-Lymphocytes in Treating Patients With Recurrent or Refractory High-Grade Malignant Glioma	1	Intravenous infusions of up to 10^8^ CAR T cells on days 1, 3, and 5 for 2 weeks. Treatment repeated after 3 weeks.	City of Hope Medical Center (National Cancer Institute)	3	**Mean survival after relapse** 11 months, positive response (Brown et al., 2015)
	**NCT01082926** Phase I Study of Cellular Immunotherapy for Recurrent/Refractory Malignant Glioma Using Intratumoral Infusions of GRm13Z40-2, An Allogeneic CD8+ Cytolitic T-Cell Line Genetically Modified to Express the IL 13-Zetakine and HyTK and to be Resistant to Glucocorticoids, in Combination With Interleukin-2	1	Intratumoral injections of 1 × 10^8^ CAR T cells and aldesleukin (IL-2) twice per week for 2 weeks.	City of Hope Medical Center	6	**Median overall survival** 19.7 months (Keu et al., 2017)

**TABLE 3:** Ongoing CAR T cell-based clinical trials in patients with GBM.

Molecular target	Clinical trial identifier and title	Study phase	CAR T cell dosage (+ combination)	Sponsor/Site (+ collaborators)	Estimated enrolment	Estimated primary completion date
**B7-H3**	**NCT04385173** Pilot Study of B7-H3 CAR-T in Treating Patients With Recurrent and Refractory Glioblastoma	1	Three intratumoral or intracerebroventricular injections of CAR T cells at two doses in between temozolomide cycles.	Second Affiliated Hospital, School of Medicine, Zhejiang University [BoYuan RunSheng Pharma (Hangzhou) Co., Ltd. (China)]	12	May, 2022
	**NCT04077866** B7-H3 CAR-T for Recurrent or Refractory Glioblastoma	1/2	Three intratumoral or intracerebroventricular injections of CAR T cells at two doses in between temozolomide cycles.	Second Affiliated Hospital of Zhejiang [Ningbo Yinzhou People’s Hospital, Huizhou Municipal Central Hospital, BoYuan RunSheng Pharma (Hangzhou) Co., Ltd. (China)]	40	June, 2024
**CD147**	**NCT04045847** CD147-CART Cells in Patients With Recurrent Malignant Glioma	1	Intracavity injection of CAR T cells, once per week for three weeks.	Xijing Hospital	31	October, 2020
**GD2**	**NCT04099797** C7R-GD2.CART Cells for Patients With GD2-expressing Brain Tumors (GAIL-B)	1	Intravenous injection of between 1x10^7^ – 1x10^8^ CAR T cells with or without lymphodepletion chemotherapy.	Baylor College of Medicine (Center for Cell and Gene Therapy, Baylor College of Medicine)	34	February, 2023
**EGFRvIII**	**NCT03726515** CART-EGFRvIII + Pembrolizumab in GBM	1	CART-EGFRvIII + pembrolizumab.	University of Pennsylvania	7	December, 2020
	**NCT03283631** Intracerebral EGFR-vIII CAR-T Cells for Recurrent GBM (INTERCEPT)	1	Starting dose of 2.5 × 10^8^ per CAR T cells per intracerebral infusion, with doses escalated in successive cohorts.	Duke University (National Cancer Institute, Duke Cancer Institute)	24	December, 2021
**IL13Ra2**	**NCT02208362** Genetically Modified T-cells in Treating Patients With Recurrent or Refractory Malignant Glioma	1	IL13Rα2-specific, hinge-optimized, 41BB/truncated CD19- expressing CAR T cells by intratumoral, intracavitary, or intraventricular catheter. Weekly for three weeks with additional infusions if eligible.	City of Hope Medical Center (National Cancer Institute, Food and Drug Administration)	92	January, 2021
	**NCT04003649** IL13Ralpha2-Targeted Chimeric Antigen Receptor (CAR) T Cells With or Without Nivolumab and Ipilimumab in Treating Patients With Recurrent or Refractory Glioblastoma	1	Intravenous administration of nivolumab and ipilimumab followed by intracranial intraventricular/intracranital intratumoral infusion of CAR T cells. Up to four cycles.	City of Hope Medical Center (National Cancer Institute)	60	December, 2022
**MMP2 (Chlorotoxin)**	**NCT04214392** Chimeric Antigen Receptor (CAR) T Cells With a Chlorotoxin Tumor-Targeting Domain for the Treatment of MPP2+ Recurrent or Progressive Glioblastoma	1	Three weekly cycles of one or two CAR T cell infusions.	City of Hope Medical Center (National Cancer Institute)	36	February, 2023
**Variable**	**NCT03423992** Personalized Chimeric Antigen Receptor T Cell Immunotherapy for Patients With Recurrent Malignant Gliomas	1	CAR T cells expressing receptors specific for EGFRvIII, IL13Rα2, Her-2, CD133, EphA2 or GD2, with or without anti- PD-L1 mAb.	Xuanwu Hospital [Beijing Mario Biotech Company, Hebei Senlang BIotech Company, Beijing HuiNengAn Biotech Company (China)]	100	January, 2021

### Targets of CAR T Cell Therapy for Which Clinical Trials Have Been Completed

#### EGFRvIII, IL13Ra2, and HER2

A deletion-mutation form of EGFR, termed EGRFvIII, is expressed in around 30% of GBM tumors (Wikstrand et al., 1997). The changes caused by this mutation to the structure of the extracellular domain provide unique epitopes which can be targeted by mAbs specific for the mutated form of EGFR, limiting the likelihood of on-target/off-tumor toxicity (Johns et al., 2004; Yang et al., 2017). In preclinical models EGFRvIII-specific CAR T cells have displayed effective tumor control (Yang et al., 2017; Chen et al., 2019); in contrast, administration of CAR T cells targeting this mutated molecule in patients with GBM has been met with limited success. O’Rourke et al. (2017) reported antigen loss and adaptive resistance in patients with recurrent GBM after intravenous injection of EGFRvIII-specific CAR T cells. A phase I/II trial (NCT01454596) utilizing CAR T cells with an anti-EGFRvIII human 139-scFv and CD28 and 4-1BB costimulatory domains in patients with malignant gliomas expressing EGFRvIII demonstrated no adverse events associated with the administration of up to 1 × 10^10^ CAR T cells. However, one patient receiving 3 × 10^10^ CAR T cells experienced serious adverse events including dyspnea and hypoxia, and another patient, who received 6 × 10^10^ CAR T cells, died 4 h post-administration after developing acute dyspnea and severe hypotension (Goff et al., 2019). These adverse events are thought to be due to a dose dependent congestion of the pulmonary vasculature by the activated T cells, likely a limitation of the route of administration utilized (Goff et al., 2019). These effects are unlikely to reflect targeting of wild-type EGFR due to cross-reactivity of the antibody used to generate the EGFRvIII-specific CAR, although this possibility cannot be ruled out. A major limitation of EGFRvIII as a target TA is its heterogeneous expression in glioma tumors, which most likely will lead to the generation of escape variants resistant to CAR T cell therapy (Rutkowska et al., 2019). These potential limitations, taken together with the low success of the first clinical trials, have markedly decreased enthusiasm for the use of this TA as a target for CAR T cells.

#### IL13Rα2

IL-13 is a cytokine released by T helper cells to regulate inflammation and immune response. Its binding to its receptor IL13Rα1 activates signaling via the JAK/STAT pathway. IL-13 can also bind to the high affinity decoy receptor IL13Rα2 which does not possess a functional cytoplasmic domain and therefore does not trigger an intracellular signaling pathway (Thaci et al., 2014). IL13Rα2 is expressed in the majority of both adult and pediatric GBM tumors but is not expressed at significant levels in normal brain or most normal tissues, with the exception of the testis (Debinski and Gibo, 2000; Kawakami et al., 2004; Jarboe et al., 2007; Brown et al., 2013; Thaci et al., 2014). Therefore it has been used as a target of CAR T cells possessing a mutated form of IL-13 in the CAR construct (Thaci et al., 2014). Administration of IL13Rα2-specific CAR T cells has been shown to be feasible and safe with encouraging clinical responses reported in a first in-human pilot study (Brown et al., 2015). Three patients with recurrent GBM received up to twelve intracranial infusions with a maximum dose of 1 × 10^8^ CAR T cells. Delivery of the IL13Rα2 CAR T cells was well-tolerated with evidence of an antitumor response of short duration in two of the three treated patients.

#### HER2

HER2 is an epidermal growth factor receptor which is expressed in normal epidermal cells. It is overexpressed on several types of cancer cells including around 80% of GBM tumors (Mineo et al., 2007). HER2 expression in normal tissues was associated with fatal toxicity in a colon cancer patient who intravenously received 1 × 10^10^ CAR T cells expressing a trastuzumab-based antigen recognition exodomain and a dual CD28.41BB.ζ signaling endodomain (Morgan et al., 2010). Subsequent analysis of the treated patient’s organs showed findings consistent with cytokine storm syndrome as well as a high accumulation of CAR T cells in normal lung and abdominal/mediastinal lymph nodes, on which HER2 is expressed, although at low level. This troubling case highlighted the need to select target TAs with limited expression on normal cells. A recent study has shown that third generation HER2-specific CAR T cells can efficiently eliminate GBM cells *in vitro* and that the activity of the administered CAR T cells is increased by their combination with PD-1 blockade (Shen et al., 2019). Successful administration of up to 1 × 10^8^ HER2-specific CAR T cells, constructed with a CD28.ζ endodomain, was achieved in GBM patients without dose-limiting toxic effects (Ahmed et al., 2017). The CAR T cells, which were generated from virus (cytomegalovirus, Epstein-Barr virus, or adenovirus) specific T cells in order to potentially provide a co-stimulatory effect through latent virus antigen recognition, were well tolerated, persisted for up to 1 year in the blood of patients and produced clinical benefit in eight out of the 17 treated patients.

The high frequency of HER2 in GBM tumors, its involvement in tumor development and progression (Hynes and MacDonald, 2009), and the ability of HER2-specific CAR T cells to eliminate both differentiated GBM cells and GBM CICs (Ahmed et al., 2010) make it an attractive target TA. The major concern when targeting HER2 are potential side effects due to HER2 expression in various normal tissues and especially in vital organs (Press et al., 1990; Morgan et al., 2010), although this has rarely been an issue with HER2-specifc CAR T cell administration in humans thus far.

## Targets of Car T Cell Therapy in On-Going Clinical Trials

### B7-H3

B7-Homolog 3 (B7-H3), also known as CD276, is a member of the B7 family of immune checkpoint molecules including PD-L1 (B7-H1) and CD80 (B7-1) (Collins et al., 2005). It is highly expressed in some hematological malignancies and in most, if not all types of solid cancer including head and neck squamous cell cancer (HNSCC), triple negative breast cancer (TNBC), intrahepatic cholangiocarcinoma (ICC), pancreatic ductal adenocarcinoma (PDAC), and chondrosarcoma (Kontos et al., 2020). Importantly it is not detectable by IHC with mono- and poly-clonal antibodies in normal tissues, with the exception of salivary glands, gastric epithelial cells and adrenal glands; in the latter normal organs its expression level is low, as suggested by the weak staining intensity. Interestingly B7-H3 is also expressed in tumor-associated vessels and fibroblasts suggesting that B7-H3 CAR T cells may eliminate cancer cells not only by direct targeting, but also through stroma disruption and neo-angiogenesis inhibition (Picarda et al., 2016; Seaman et al., 2017). The described features make B7-H3 a highly attractive target of antibody-based immunotherapy. The efficacy of B7-H3 CAR T cell-based therapy has been explored in a wide range of cancer types including neuroblastoma, pancreatic ductal adenocarcinoma and ovarian cancer (Du et al., 2019; Majzner et al., 2019). Tang et al. (2019) have recently assessed the antitumor effect of B7-H3 CAR T cells in intracranial GBM mouse models, and concluded that they could induce a significant tumor regression and prolong survival of tumor bearing mice compared to vehicle-transduced T cells. A randomized, parallel-arm, phase I/II study (NCT04077866) has been designed to assess the effect of B7-H3 CAR T cell administration between cycles of Temozolomide treatment for patients with refractory or recurrent GBM.

A potential concern, however, is that B7-H3 mRNA is present practically in all normal tissues, although it is not translated due to inhibition by microRNAs (Xu et al., 2009). This inhibition can easily be modulated by conditions such as inflammation, resulting in B7-H3 expression in normal tissues, which then may be targeted by B7-H3 CAR T cells.

### CD147

CD147, also known as extracellular matrix metalloproteinase inducer (EMMPRIN), is a 57-kilodalton (kDa) type-I transmembrane protein belonging to the immunoglobulin superfamily of adhesion molecules. It induces fibroblasts to secrete metalloproteinases-1, -2, -3, -9, -14, and -15, which degrade the extracellular matrix (ECM), promoting tumor growth, invasion and metastasis (Xiong et al., 2014). CD147 expression has been found to be significantly higher in glioma than in normal brain tissues; its expression level is inversely correlated with prognosis in patients with GBM (Yang et al., 2013; Li et al., 2017). An open label early phase I clinical trial has started to recruit patients with recurrent GBM to test the safety, tolerance and efficacy of treatment with CD147-specific CAR T cells (NCT04045847). While CD147 is overexpressed on malignant cells, it is still expressed at a low level on various normal tissues such as epithelial and endothelial cells, and brain and heart tissue (Riethdorf et al., 2006; Liao et al., 2011; Tseng et al., 2020). Therefore, there is a concern that CD147-specific CAR T cells may cause on-target off-tumor side effects.

### GD2

Gangliosides are commonly expressed on normal tissues. Interestingly disialoganglioside GD2 is not, but is highly expressed on several tumor types including melanoma, retinoblastoma and neuroblastoma (Nazha et al., 2020). GD2 is expressed on GBM cell lines and patient samples making it an attractive target TA (Golinelli et al., 2018). Furthermore, GD2 has been reported to be a CIC marker in breast cancer (Battula et al., 2012), however, this finding is questionable for GBM as Woo et al. (2015) found that patient-derived GBM cells with or without GD2 had similar *in vitro* neurosphere formation capacity.

GD2 targeting CAR-T cells have successfully demonstrated potent cytotoxicity against neuroblastoma cell lines *in vitro* as well as against cell lines grafted subcutaneously in NOD/SCID mice models (Prapa et al., 2015). Additionally, GD2-specific CAR T cells are able to effectively eliminate patient-derived diffuse midline glioma orthotopic xenograft models (Mount et al., 2018). In an alternative approach, the anti-tumor activity of TNF-related apoptosis-inducing ligand (TRAIL) expressing mesenchymal stromal/stem cells (MSCs) have been used to eliminate cancer cells (Grisendi et al., 2015). The addition of a truncated GD2-specific CAR to the MSCs enhanced specific targeting of GD2-positive GBM cells *in vitro* (Golinelli et al., 2018).

GD2-specific CAR T cells have been safely administered to patients with neuroblastoma, albeit with limited clinical benefit (Louis et al., 2011; Heczey et al., 2017). Together this information has led to the initiation of a clinical trial targeting high grade gliomas including GBM (NCT04099797). In this trial GD2-specific CAR T cells were also transduced to express a constitutively active IL-7 cytokine receptor to enhance their antitumor activity (Shum et al., 2017).

### MMP2

Chlorotoxin (CLTX) is a small, naturally derived 36–amino acid long peptide found in the venom of the death stalker scorpion Leiurus quinquestriatus (DeBin et al., 1993). Interestingly CLTX binds selectively to primary brain tumors, but displays barely detectable binding to normal brain tissue as well as many other normal human tissues tested including skin, kidney and lung (Lyons et al., 2002). The specific surface receptor for CLTX on GBM cells has not been identified, however, the expression of matrix metalloproteinase 2 (MMP2), chloride channel CLCN3, and phospholipid protein annexin A2 (ANXA2) all appear to be required for CLTX binding to GBM cells (Soroceanu et al., 1998; Deshane et al., 2003; McFerrin and Sontheimer, 2006; Tatenhorst et al., 2006). CLTX bioconjugate administration for imaging and therapeutic purposes has been well tolerated in patients with no dose limiting toxicity observed (Cohen et al., 2018). CLTX-directed CAR T cells generated to target GBM cells showed potent antitumor activity in orthotopic xenograft tumor models (Wang et al., 2020). GBM cells in which MMP2 expression was knocked down using short hairpin RNA (shRNA) resulted in significantly lower CLTX CAR T cell activation and cytotoxicity (Wang et al., 2020). These results imply that membrane associated MMP2 is required for effective CLTX CAR T cell targeting of GBM cells. In light of this information a phase I study (NCT04214392) for the treatment of MMP2 positive recurrent or progressive GBM with T cells expressing CLTX CARs has been initiated.

### NKG2D Ligands

The human NKG2D receptor is expressed by the majority of natural killer (NK) cells of the innate immune system as well as by NKT, γδ T cells, CD8+ T cells, and some autoreactive or immunosuppressive CD4+ T cells (Duan et al., 2019). NKG2D ligands, which include MHC class I related chains (MICA/B) and six UL16-binding proteins (ULBPs), are often upregulated on stressed, transformed and pathogen-infected cells; therefore, they play a crucial role in their detection and elimination by effector immune cells (Bauer et al., 1999; Raulet et al., 2013). NKG2D ligands are expressed on GBM cell lines, patient samples and importantly on GBM stem-like cells (Flüh et al., 2018; Yang et al., 2019). Treatment with chemotherapy or radiotherapy can upregulate NKG2D ligand expression on GBM cells, emphasizing the potential of combinatorial therapeutic strategies (Weiss et al., 2018a). CAR T cells expressing full length murine NKG2D in combination with radiotherapy have been reported to significantly prolong overall survival of immunocompetent mice with intracranial grafts of mouse glioma cells (Weiss et al., 2018b). Furthermore, Yang et al. (2019) have demonstrated effective eradication of human differentiated GBM cells and GBM CICs *in vitro* and in subcutaneous tumor models. However, toxicity in humans cannot be excluded given the expression of NKG2D ligands on normal tissues under distress. This information should have been provided by a phase I clinical trial to test the safety and clinical response to NKG2D-based CAR T cells in solid tumors including GBM (NCT04270461). However, this trial has been withdrawn for administrative reasons.

## Targets of Car T Cell Therapy in Preclinical Studies

### CAIX

Carbonic anhydrases (CA) are a group of enzymes which catalyze the reversible hydration of carbon dioxide, a process which is important for several cellular functions including maintenance of pH balance (Supuran, 2016). CAIX is induced under hypoxic conditions and is therefore often overexpressed in many solid tumors including GBM (Proescholdt et al., 2005), for which it is a prognostic marker of poor patient survival outcome (Proescholdt et al., 2012). CAIX-specific CAR T cells have been tested against GBM cells *in vitro* and following direct intratumoral injection in an *in vivo* xenograft mouse model (Cui et al., 2019). The CAIX-specific CAR T cells effectively eliminated GBM cells and prolonged survival of tumor bearing mice. However, the regulation of CAIX expression level on cancer cells by the degree of hypoxia in the TME is likely to cause marked changes in its expression on cancer cells. Therefore, there may be a significant degree of intra- and inter-tumor heterogeneity in CAIX expression in a patient. This expression pattern is likely to facilitate the generation of cancer cell escape variants, which may represent a major obstacle to the successful application of CAIX-specific CAR T cell-based therapy.

### CD70

CD70 is a type II transmembrane protein binding only to CD27, a glycosylated transmembrane protein of the tumor necrosis factor (TNF) receptor family. Besides being expressed on activated B and T cells and mature DCs, CD70 is expressed on certain hematological and solid cancers including GBM. Constitutive CD70 expression may provide GBM cells with an immune escape mechanism by promoting T cell death (Chahlavi et al., 2005). Jin et al. (2018) have recently reported that both human and mouse CD70-specific CAR T cells could recognize and eliminate CD70+ GBM tumors *in vitro* and in xenograft and syngeneic models, with no toxicity reported. A major strength of this TA as a target is its restricted expression mostly to malignant tumors, both primary and recurrent lesions; Jin et al. (2018) also showed that CD70 gene expression was not detected in 52 types of normal tissues. While there could be a concern related to targeting of CD70+ T cells and DCs, CD70-specific CAR T cells do not seem to affect these activated immune cells. Preclinical studies utilizing CD70-specific CAR T cells in glioma and head and neck cancer (Park et al., 2018) have shown encouraging results and provide enthusiasm for the targeting of this TA.

### CSPG4

Chondroitin Sulfate Proteoglycan 4 (CSPG4), also known as neuron-glial antigen 2 (NG2) and high molecular weight-melanoma associated antigen (HMW-MAA), was initially identified on melanoma cells, but has since been shown to be expressed by a wide variety of cancer types, as well as on CICs in many of the cancer types analyzed (Campoli et al., 2010). It is thought to play a role in cell proliferation and migration *in vitro* as well as in metastatic spread *in vivo*; its expression level is inversely correlated with patient survival in glioma (Tsidulko et al., 2017) as well as in other solid cancers such as HNSCC and chordoma (Wang et al., 2010b; Warta et al., 2014; Schoenfeld et al., 2016). CSPG4 expression, assessed via commercially available antibodies, has been reported in the Protein Atlas to have a broad distribution in normal tissues. However, IHC staining with our mAbs which recognize distinct CSPG4 epitopes has not detected them in any normal tissue with the exception of activated pericytes in the TME (Campoli et al., 2010). Furthermore, negative reverse protein assay results derived from the analysis of 94 normal tissues (Beard et al., 2014), lack of toxicity in CSPG4-specific mAb treated animal models (Mittelman et al., 1992; Wang et al., 2010a; Riccardo et al., 2014) and inability of CSPG4-specific CAR T cells to recognize and lyse normal cells *in vitro* (Geldres et al., 2014) convincingly argue against the possibility that CSPG4 expression on normal tissues is an issue when targeting this molecule as a TA. This conclusion is supported by the following additional lines of evidence: no toxicity was detected in melanoma patients who developed CSPG4-specific antibodies after immunization with anti-idiotypic antibodies which mimic CSPG4 (Mittelman et al., 1992). Furthermore Pellegatta et al. (2018) recently demonstrated that CSPG4 is highly expressed with limited heterogeneity in GBM tissue and tumor associated vessels, and is not detected in healthy brain parenchyma. Intracranial delivery of CSPG4 CAR T cells was able to control tumor progression in orthotopic GBM neurosphere xenograft models (Pellegatta et al., 2018). The encouraging preclinical results, the lack of expression in normal brain tissue and the involvement in several processes of tumorigenesis make CSPG4 a very attractive target TA. Furthermore, its expression on CICs will enable CAR T cells to recognize and eliminate this particularly hostile cell subpopulation.

### EphA2

Erythropoietin-producing hepatocellular carcinoma A2 (EphA2) belongs to the Eph family of receptor tyrosine kinases (RTKs); it plays a role in a wide variety of functions in malignant cells, such as tumorigenesis, invasion, angiogenesis and metastasis (Wykosky and Debinski, 2008; Baharuddin et al., 2018). Therefore, targeting EphA2 could prevent tumor progression and recurrence. EphA2 is overexpressed in GBM with no detection in normal brain tissues (Hatano et al., 2005; Wykosky et al., 2005). It has also been found to be expressed on GBM CICs (Binda et al., 2012). EphA2 targeting CAR T cells effectively eliminate differentiated GBM cells and GBM cancer stem-like cells *in vitro*, and significantly prolong survival of orthotopic xenograft SCID mouse models (Chow et al., 2013). In a subsequent study Yi et al. (2018) generated an optimized version of the anti-EphA2 CAR construct utilizing a short spacer region. This CAR displayed greater anti-glioma activity compared to the original CAR since survival of glioma bearing mice was prolonged to a similar extent using a 20-fold lower dose (Yi et al., 2018).

### TROP2

Trophoblast cell surface antigen 2 (TROP2) is a 36 kDa transmembrane glycoprotein, highly expressed on several types of solid cancer (Cubas et al., 2009; Zeng et al., 2016). It is also considered a stem cell marker (Lenárt et al., 2020). Hou J. et al. (2019) demonstrated high TROP2 expression on GBM cells from surgically removed patient tumors compared to low levels on normal brain cells. Its expression level was found to inversely correlate with survival in GMB patients (Hou J. et al., 2019). TROP2 is thought to modulate cell proliferation and promote metastasis via activation of JAK/STAT3 pathway (Hou J. et al., 2019). Furthermore, in GBM TROP2 is associated with promotion of blood vessel formation through VEGF upregulation (Hou J. et al., 2019). Therefore, targeting TROP2 may also help to inhibit cancer growth through abrogation of neoangiogenesis.

In a recent study, Bedoya et al. (2019) have shown that TROP2-specific CAR T cells can target TROP2 expressing breast, pancreas and prostate cancer cells highlighting the potential of this TA to mediate the targeting of different types of solid cancer. GBM cell recognition and elimination by TROP2-specific CAR T cells is under investigation. Although the latter results suggest that TROP2 may be a promising target, its wide expression on various healthy tissues (Li et al., 2020) raises concerns about the induction of on-target off-tumor toxicity, which could potentially greatly limit its clinical application.

## Limitations of Car T Cell Therapies to Treat GBM

Whilst CAR T cells targeting CD19+ hematological malignancies have shown remarkable clinical responses resulting in FDA approval of tisagenlecleucel (KYMRIAH, Novartis), axicabtagene ciloleucel (YESCARTA, Kite Pharmaceuticals) and brexucabtagene autoleucel (TECARTUS, Kite Pharmaceuticals) CAR T cell therapies for clinical use (Beyar-Katz and Gill, 2020), this success has not yet been paralleled in solid tumors. Several challenges face the application of CAR T cell therapy in solid tumors; they include: (i) insufficient trafficking of CAR T cells to the tumor site, (ii) defective recognition of the targeted TA, (iii) expression of the targeted TA in normal tissues, leading to killing of normal cells, (iv) limited persistence and low proliferation of effector immune cells in the TME, (v) unregulated strength and timing of effector functions resulting in adverse effects such as those caused by cytokine release syndrome and, (vi) immunosuppressive TME which provides multiple escape mechanisms to tumor cells (Lim and June, 2017). These variables and their role have been extensively reviewed elsewhere (June and Michel Sadelain, 2018; June et al., 2018; Schmidts and Maus, 2018; Martinez and Moon, 2019). Here we focus on issues specifically affecting CAR T cell treatment of GBM (Chuntova et al., 2021) such as acquired resistance, penetration of blood brain barrier (BBB) and toxicity of central nervous system (CNS).

### Acquired Resistance

Gliomas are characterized by genetic, epigenetic, and environmental intratumoral heterogeneity (Nicholson and Fine, 2021). Intratumoral TA heterogeneity and antigen/epitope loss following treatment are potential causes of failure of CAR T cell therapies in GBM. EGFRvIII TA escape has previously been observed following the use of an EGFRvIII targeted peptide vaccine in GBM patients; 82% of the patients who had disease recurrence had lost the targeted TA (Sampson et al., 2010). Similarly EGFRvIII loss/downregulation has been reported following EGFRvIII-specific CAR T cell administration (O’Rourke et al., 2017). While peripheral blood engraftment and persistence of CAR T cells could be detected in patients with disease controlled in the short term, surgically resected tumors had EGFRvIII loss or downregulation. A similar observation has been made following IL13Rα2 CAR T cell administration (Krenciute et al., 2017). The resulting resistance is thought to be due to the survival and growth of differentiated GBM cells and/or GBM CICs which escape CAR T cell-mediated killing because of the lack of expression of the targeted TA and progress the disease with an altered phenotype. Therefore, selection of a TA with high homogenous expression and high expression stability, or development of methods to enhance the ability of CAR T cells to eliminate cancer cells which do not express the targeted TA are necessary to prevent this cancer escape mechanism.

### Blood Brain Barrier

The BBB is a physiological barrier between the blood vessels carrying oxygen and nutrients and the brain tissues they supply. The structure of the BBB is complex, but primarily consists of specialized endothelial cells very tightly joined to each other in contact with pericytes and astrocytes. This morphology helps isolate brain from disease-causing pathogens and toxins and was historically thought to help make the brain an ‘immune privileged organ’ (Carson et al., 2006). This view has changed due to the recognition that peripheral immune cells can cross the intact BBB, allowing brain to interact with immune system. However, BBB does limit leukocyte migration into CNS and therefore regulates the rate of T cell recruitment (Engelhardt, 2010). Cell adhesion molecules on endothelial cells as well as specific antigen expression by APCs are thought to be necessary to recruit antigen-specific CD8+ T cells across BBB (Galea et al., 2007; Engelhardt, 2010). However, T cell recruitment in the presence of cancer is often reduced (Sackstein et al., 2017). This reduction may provide cancer cells with an immune escape mechanism.

Glioblastoma multiforme was previously suggested to uniformly damage BBB; therefore, permeability of drugs, antibodies and immune cells should not be an obstacle. However, it has been recently demonstrated that BBB may be intact in spite of GBM even with significant tumor burden (Sarkaria et al., 2018). Therefore, the mode of delivery of CAR T cells to treat GBM should be reevaluated.

Systemic intravenous administration of CAR T cells results in limited CAR T cell infiltration of tumors located in the CNS (Mulazzani et al., 2019). Intravenous administration of EGFRvIII-specific CAR T cells to treat GBM displayed some infiltration of CAR T cells into tumor, although this was not consistent in all patients (O’Rourke et al., 2017). To overcome this limitation, direct administration of CAR T cells to the tumor site has been assessed as a mode of delivery, negating the need for cells to migrate across the BBB. This can either be achieved by intratumoral/intracavitary injection into the tumor or the resected tumor site, or by intracerebral/intraventricular injection into the brain tissue or cerebral ventricle. Locoregional administration of CAR T cells has been found to improve T cell tumor infiltration and tumor control in several preclinical models of brain tumors, compared to intravenous delivery (Priceman et al., 2018; Mulazzani et al., 2019; Theruvath et al., 2020). Intracerebral CD19-specific CAR T cell injection to mice with CNS lymphoma was found to result in the migration of CAR T cells to the ‘healthy’ contralateral brain hemisphere, albeit at lower numbers (Mulazzani et al., 2019). Similarly in a murine GBM model intraventricular administration of IL13Rα2-specific CAR T cells was found to convey greater control of the contralaterally grafted tumor (Brown et al., 2018). This finding is of great clinical relevance as multifocal GBM accounts for nearly a quarter of primary GBMs and therefore targeting of all tumor sites is crucial to cure a patient (Syed et al., 2018). Furthermore, in a recent case report a multifocal GBM patient initially received IL13Rα2 CAR T cells via intracavitary infusion to the resected tumor site, which was able to control locoregional progression, but not that of non-resected tumors in the contralateral temporal lobe or the development of new tumors (Brown et al., 2016). Interestingly, once the administration method was changed to intraventricular infusions into the opposite lateral ventricle, all intracranial and spinal tumors were reduced in size. Therefore, intraventricular infusion of CAR T cells appears to be the most effective delivery method to administer CAR T cells to all tumor sites, as demonstrated by its current use in most ongoing clinical trials (Table 3).

### Toxicity Associated With CAR T Cell Treatment in GBM

Cytokine release syndrome (CRS) is the most common adverse event associated with CAR T cell therapy usually seen within one or 2 weeks after the initial infusion (Lee et al., 2014; Bonifant et al., 2016). Substantial activation of CAR T cells can cause the release of an excessive amount of inflammatory cytokines which can subsequently result in fever, tachycardia, hypotension and in some cases even death due to multiple organ failure (Schuster et al., 2017).

A major concern in the administration of CAR T cells to the brain is the potential for neurotoxicity, which can occur alone or together with CRS. The mechanism(s) underlying CAR T cell mediated neurotoxicity has not been elucidated yet; however, CNS endothelial cell activation is thought to play a role (Mackall and Miklos, 2017; Wang and Han, 2018). Gust et al. (2017) showed that CD19-specific CAR T cell administration for the treatment of B cell acute lymphoblastic leukemia increased permeability of BBB as a result of endothelial cell activation. The latter, in turn, leads to a cytokine influx into CNS causing a range of side effects such as seizures and cerebral edema, resulting in several fatalities.

Therefore, CAR T cell administration directly to brain raises some concerns about the risks of CRS and neurotoxicity occurring in this sensitive organ. CAR T cell treatment of GBM has resulted so far in only one such fatality of a patient who received the highest number of EGFRvIII CAR T cells in a dose escalation phase I trial (Goff et al., 2019). Aside from this episode most trials of GBM CAR T cell therapy have shown relatively few adverse events.

To decrease CAR T cell toxicity, strategies to inhibit excessive cytokine release are under investigation. These include (i) administration of high dose steroids, and/or of a mAb (tocilizumab) targeting the IL-6 receptor, (ii) selection of optimal CAR T cells in terms of binding avidity and/or antitumor activity, (iii) administration of lower numbers of CAR T cells per infusion, and (iv) utilization of CAR T cells with a suicide gene or switchable signaling components.

### Need to Eliminate CICs

Cancer initiating cells are a subpopulation of cells within a tumor, which according to the cancer stem cell theory (Reya et al., 2001) play a crucial role in cancer initiation and metastatic spread due to their characteristics of self-renewal and multi-lineage differentiation. In GBM CICs are defined by the expression of intracellular markers usually associated to stem cells such as MYC, NANOG, and SOX2 and of cell surface markers including CD133 (Lathia et al., 2015). However, a single diagnostic marker has not been identified yet and indeed GBM CICs may represent a more plastic state of cancer cells (Dirkse et al., 2019). Alternatively, but not exclusively GBM CICs can be detected by their ability to form neurospheres *in vitro* (Lathia et al., 2015) and to induce tumors when injected in low numbers to immunodeficient mice (Lathia et al., 2015). CICs have been suggested to be partially responsible for treatment failure and disease recurrence due to their self-renewal ability and treatment resistance. Ability of GBM sphere generation *in vitro* using dissociated patient tumor samples as well as the number of CD133+/Ki67+ cells in the lesion are both prognostic markers of tumor progression and poor patient survival outcome (Pallini et al., 2008). These findings emphasize the need to develop and implement therapeutic strategies that efficiently eliminate the CIC subpopulation.

CD133-specific CAR T cells have been shown to successfully eliminate GBM CICs in an orthotopic *in vivo* model (Zhu et al., 2015). CAR T cells targeting B7-H3, CSPG4 and HER2 have also been shown to eliminate both differentiated GBM cells and GBM CICs in preclinical models of GBM (Ahmed et al., 2010; Pellegatta et al., 2018; Nehama et al., 2019). Taken together these results provide evidence of the efficacy of CAR-engineered T cells in targeting both differentiated GBM cells and GBM CICs.

## Strategies to Enhance Efficacy of Car-Based Immunotherapy Against GBM

In many clinical studies CAR T cells used as a monotherapy have been found not to be sufficient to induce sustained clinical responses in various types of solid cancers (Hou B. et al., 2019). In GBM the reported clinical trials have displayed limited clinical efficacy with one trial utilizing EFGRvIII-specific CAR T cells terminated in order to pursue combination therapy with Pembrolizumab (NCT03726515). One might argue that the lack of clinical response to therapy with CAR T cells alone is partially due to recruitment of patients at later stages of disease with high tumor burden and a history of unsuccessful therapies. These disappointing clinical results highlight the need to develop novel strategies to increase CAR T cell antitumor activity and persistence (Xu J. et al., 2018).

### Enhancing Functional Properties of CAR T Cell Constructs

Successive generations of CAR T cells have enhanced the strength and potency of their antitumor activity through the addition of co-stimulatory domains and functionality moieties. Current CAR T cells in clinical trials are utilizing these approaches such as the chlorotoxin (EQ)-CD28-CD3zeta/truncated CD19-expressing CAR T cells and IL13Rα2-specific hinge-optimized 4-1BB/truncated CD19-expressing CAR T cells. Interestingly both of these CAR T cells incorporate a truncated version of CD19 which has no functional activity, but can be utilized as a marker to identify transduced CAR T cells.

One mechanism to enhance antitumor efficacy is to further engineer the CAR construct to induce or constitutively secrete active cytokines in order to increase CAR T cell activity and persistence. IL13Rα2 CAR T cells engineered to additionally express IL-15 displayed greater anti-glioma activity and improved persistence and significantly prolonged survival of mice than control IL13Rα2 CAR T cells in orthotopic glioma xenograft models (Krenciute et al., 2017). Several mechanisms designed to disrupt immunosuppressive immune checkpoint molecule signaling between CAR T cells and malignant cells have been tested (Chen et al., 2016). CAR T cells engineered to secrete a PD-L1 antibody (Suarez et al., 2016), CAR T cells with PD-1 and Lag3 genes knocked out using CRISPR/Cas9 technology (Ren et al., 2017; Zhang et al., 2017), and CAR T cells designed with a PD-1 ectodomain linked to the transmembrane and cytoplasmic domains of CD28 in order to convert an immunosuppressive signal into a co-stimulatory one (Prosser et al., 2012; Liu et al., 2016) have all been explored in several solid cancer types. In preclinical GBM models, use of EGFRvIII-specific CAR T cells in which PD-1 signaling had been disrupted through a CRISPR-Cas9 approach, resulted in significantly prolonged survival of the orthotopically engrafted mice (Choi et al., 2019c).

Similarly, CAR T cells can be engineered to express chemokine receptors to enhance intra tumoral T cell trafficking. CXCR1 and CXCR2 modified CD70-specific CAR T cells were shown to have improved T cell trafficking and antitumor efficacy through IL-8 mediated chemotaxis in *in vivo* models of GBM (Jin et al., 2019). The higher CAR T cell antitumor activity resulted in improved tumor regression and survival of mice compared to those treated with unmodified CD70-specific CAR T cells. Additional approaches to enhance CAR T cell antitumor activity include incorporation of the hypoxia transcription factor HIF-1α subdomain in a CAR construct. This results in the activation of CAR T cells only under hypoxic conditions such as in the TME (Juillerat et al., 2017). This strategy may reduce on target/off tumor toxicity. Cancer cells often secrete adenosine which can inhibit T cell activity within the TME (Ohta, 2016). Blockade of the A2A adenosine receptor using pharmacological antagonists or target specific shRNA can increase CAR T cell efficacy in orthotopic models of breast cancer (Beavis et al., 2017). However, blockade of the A2A adenosine receptor may be counterproductive in GBM as the FDA-approved A2A adenosine receptor agonist lexiscan has been shown to increase BBB permeability in *in vitro* and *in vivo* models (Kim and Bynoe, 2015).

Tumor antigen expression on normal tissues often hinders the usage of CAR T cells due to the concern of unexpected side effects and toxicity that will be seen in healthy tissues as a result of the treatment. To overcome this challenge CARs designed using scFvs with altered affinity allows differential recognition of the targeted TA which are highly expressed on cancer cells but have a lower expression level on normal tissues. For example CARs targeting HER2 or EGFR with reduced affinity demonstrated effective elimination of cancer cells with no damage to normal cells both *in vitro* and in ovarian and prostate murine models, and therefore may be useful in GBM treatment (Liu et al., 2015).

### Targeting Multiple TAs

As TA expression is frequently heterogeneous on GBM tissues, as on other types of solid cancer tissues, malignant cells lacking the targeted TA may escape CAR T cell recognition and elimination. Recurrences of the disease have been observed due to outgrowth of cancer cells that do not express the targeted TA after CAR T cell treatment (Krenciute et al., 2017; O’Rourke et al., 2017). One strategy to overcome this escape mechanism relies on targeting multiple TAs at the same time (Grada et al., 2013). For instance, CAR T cells which target multiple ligands such as NKG2D and ErbB have been investigated for their efficacy against solid tumors both *in vitro* and *in vivo*, and have displayed effective antitumor results (Davies et al., 2012; Sentman and Meehan, 2014). Another strategy is to incorporate multiple antibody scFvs in the same CAR T cell construct. Bi-specific CAR T cells expressing both HER2 and IL13Rα2 displayed increased tumor elimination compared to singular TA-specific CAR T cells in a murine model of GBM (Hegde et al., 2016). Similarly, CAR T cells specific for both CD70 and B7-H3 have shown effective preclinical antitumor function against a range of solid tumors (Yang et al., 2020). Furthermore, tri-specific CAR T cells targeting HER2, IL13Ra2, and EphA2 provide an even more comprehensive coverage of TAs and have been shown to significantly prolong survival of mice bearing GBM patient derived xenografts (Bielamowicz et al., 2018). An alternative approach involves the use of EGFRvIII-specific CAR T cells engineered to secrete bi-specific T-cell engagers (BiTE) (Choi et al., 2019b). These bi-specific mAbs can link T cells to wild type EGFR, overcoming the resistance of EGFRvIII heterogenous GBM to EGFRvIII-specific CAR T cells. BiTE-armored CAR T cells successfully eliminated cancer cells and prolonged survival of mice orthotopically grafted with either GBM cell lines or patient derived glioma neurospheres (Choi et al., 2019b). In order to improve the antitumor effect of bi-specific CAR T cells their signaling requirements may have to be optimized (Han et al., 2019). Activation of bi-specific CAR T cells could be triggered by only one of the targeted TAs or may require the expression of both TAs on target cells. Alternatively, inhibition of bi-specific CAR T cells could be triggered by recognition of a selected target antigen expressed on non-malignant cells to minimize on-target/off-tumor toxicity.

### CAR NK Cells as Effectors for the Treatment of GBM

An alternative therapeutic strategy to CAR T cells is to incorporate a CAR construct into other types of effector immune cells such as NK cells (Burger et al., 2019). In particular NK cells, usually considered a component of the innate immune system, offer additional advantages over T cells and CAR effector cells in that they can (i) recognize multiple non-CAR specific oncogenic antigens, (ii) be administered as an allogeneic transplant with low risk of graft versus host disease, (iii) regulate adoptive immune responses though DC editing, and (iv) do not induce CRS. However, major limitations of CAR NK cells are the relatively low yield of NK cells available from an individual donor’s peripheral blood compared to T cells, as well as the low transduction efficiency of CAR constructs in these cells. Strategies to expand NK cell populations isolated from the blood of a donor, as well as use of NK cell lines to create an ‘off the shelf’ CAR NK cell product are highly promising approaches to overcome these limitations.

Preclinical studies have demonstrated the potent antitumor activity of CAR NK cells recognizing several TAs including EGFR and EGFRvIII. The NK cell line NK-92 has been utilized most prominently to successfully target these TAs on GBM cells in *in vitro* culture assays and in orthotopic *in vivo* models (Han et al., 2015). Notably, strategies to improve CAR NK cell antitumor activity have also been assessed including additional co-expression of the chemokine receptor CXCR4 to promote homing to the tumor site (Müller et al., 2015) and dual EGFR and EGFRvIII targeting CAR NK cells (Genßler et al., 2016). Both of these strategies demonstrated improved tumor control in NSG mice harboring orthotopic GBM xenografts. HER2-specific CAR NK cells have displayed antitumor activity against GBM cells *in vitro* and in a GBM xenograft mouse model (Zhang et al., 2016).

This convincing preclinical data has led to implementation of the CAR2BRAIN phase I clinical trial (NCT03383978) treating recurrent or refractory HER2-positive GBM patients with HER2-specific CAR NK cells derived from the NK-92 cell line (Burger et al., 2016). As murine models indicate that intravenously administered NK-92 cell line derived CAR NK cells cannot cross the BBB without ultrasound disruption (Alkins et al., 2013), intracranial injection of cells into the wall of the resection cavity has been selected as the route of administration. No results are available yet. Several other clinical trials using NK-92 derived CAR NK cells are ongoing in China to treat various cancer types, demonstrating the feasibility and overall safety of this approach (Tang et al., 2018; Zhang et al., 2019). A major limitation of using NK cell lines compared to primary effector cells, however, is the need to irradiate them prior to infusion in order to abrogate the possibility of secondary malignancy formation. As a result, NK cells cannot engraft or expand *in vivo* and therefore have a limited activity timespan. The results of clinical trials are needed to assess the efficacy of CAR NK cell treatment of GBM.

### Universal CAR T Cells

The future of CAR T cell engineering lies in overcoming reliance on selective TAs and allowing them the flexibility to target several TAs, and the capacity to be turned on or off in a timely and effective manner to avoid toxicity. Several mechanisms to reach this goal are in development. T cells with CARs specific for antibodies such as FITC-tagged mAbs can effectively target cancer cells *in vitro* and *in vivo* using FITC-conjugated mAbs, but display minimal functionality in the presence of unlabeled mAbs (Tamada et al., 2012). Similarly, T cell constructs expressing CD16 (CD16-CR T cells) and CD32 (CD32-CR T cells) to target cancer cells through the addition of TA-specific mAbs and induction of antibody dependent cellular cytotoxicity have been explored (Caratelli et al., 2017). More recently, split universal and programmable (SUPRA) engineered CAR T cells, which link an intracellular signaling domain to a leucine zipper extracellular domain have been generated. Following addition of zipFv adaptor molecules containing a leucine zipper that will bind to the extracellular domain of the CAR, and a ligand binding domain specific for a selected TA, the CAR T cell is then able to recognize and eliminate target cells (Cho et al., 2018).

The advantages of this type of CAR T cells are the broad cancer applicability, capacity to overcome TA loss and ability to regulate functional effects. They therefore are attractive candidates to be tested in GBM.

## Combinatorial Therapy to Enhance Car T Cell Efficacy

Whilst enhancement of a CAR construct can improve the antitumor activity of the CAR effector cell it may not be sufficient to overcome the limitations of the immunosuppressive TME. Combining CAR T cells with other therapeutic strategies can produce an additive or a synergistic effect enabling functional CAR T cells to recognize and eliminate cancer cells which they otherwise were unable to. Whilst there are many therapeutic strategies being investigated in combination with CAR T cells such as epigenetic drugs and kinase inhibitors against various cancer types, here we review those currently being explored in GBM.

### Combination With Chemo-/Radio-Therapy

As standard therapeutic options for GBM, chemotherapy and radiotherapy are theoretically easily applicable therapies that could be used in combinatorial strategies with CAR T cells. It is thought that both chemotherapeutic agents and radiotherapy may sensitize cancer cells to elimination by CAR T cells through several mechanisms.

Radiotherapy can induce changes in the TME that may help boost CAR T cell efficacy (Flynn et al., 2017; Minn et al., 2019). Firstly, radiation can upregulate the expression of many types of TAs on malignant cells. This phenotypic change can result in enhanced malignant cell recognition and elimination by cognate cytotoxic CD8^+^ T cells (Reits et al., 2006; Zhang et al., 2007; Jin et al., 2018). Secondly, radiation can increase infiltration of a tumor by immune cells because of the release of proinflammatory cytokines such as IFN-γ (Lugade et al., 2008) as well as chemokine ligands which can recruit T cells to the TME (Lugade et al., 2008; Matsumura et al., 2008). Furthermore, radiation may structurally alter the TME, disrupting the established vasculature as well as increasing the permeability of the BBB (Portella and Scala, 2019; Arvanitis et al., 2020). Radiation also induces tumor necrosis and apoptosis which can trigger the release of danger signals. The latter in turn can induce type I IFN production and increase the maturation and activation of DCs which can improve TA presentation and lead to a more effective endogenous immune response (Burnette et al., 2011; Crouse et al., 2015). This endogenous immune response can contribute to an abscopal effect which has been reported following radiotherapy and may act synergistically with the CAR T cell therapy (Demaria et al., 2004). Combination with radiotherapy has been found to improve efficacy of CAR T cell therapy in models of GBM as well as some other solid tumors (DeSelm et al., 2018; Jin et al., 2018; Weiss et al., 2018b). Jin et al. (2018) reported that CD70 expression is upregulated on GBM cells following irradiation and that this enhanced CD70-specific CAR T cell mediated tumor cell elimination. Similarly Weiss et al. (2018b) reported increased immune cell infiltration and activity of CAR T cells when combining radiotherapy and NKG2D CAR T cells in murine GBM models.

Chemotherapy provides similar mechanistic changes to the TME as radiotherapy which may enhance CAR T cell efficacy including TA upregulation (Zhang et al., 2007) and immunosuppressive cell elimination (Lutsiak et al., 2005). Furthermore conditioning chemotherapy enhances the persistence and expansion of adoptively transferred T cells, improving the efficacy of CAR T cells (Muranski et al., 2006; Xu J. et al., 2018). Therefore, optimization of the most suitable way to combine chemotherapy and CAR T cells is being explored in several of the current GBM clinical trials.

### Combination With Immune Checkpoint Blockade

Perhaps the most promising combinatorial strategy to be investigated is the synergistic application of CAR T cells with immune checkpoint blockade (ICB) therapy. These drugs block the inhibitory signaling pathways utilized by tumor cells to dampen immune effector cell activity (Korman et al., 2006; Sharma and Allison, 2015). Their use as a monotherapy has shown significant clinical benefit in a proportion of patients with malignant melanoma, lung cancer and renal cell cancer (Topalian et al., 2019), however, results from early trials in GBM patients have not shown significant survival benefit (Romani et al., 2018; Desai et al., 2019). The use of ICB as a monotherapy is limited by the need of a cognate T cell response and presentation of neoantigens by HLA class I antigens on cancer cells. The addition of CAR T cells specific for the tumor can overcome these requirements. Checkpoint inhibitors targeting PD-1/PD-L1 and CTLA4 pathways have been shown to increase the activity of CAR T cells in preclinical models of GBM (Shen et al., 2020). Currently the clinical trial NCT04003649 is investigating whether IL13Ra2 CAR T cells work better alone or in combination with nivolumab (anti-PD-1) and ipilimumab (anti-CTLA4) to treat recurrent and refractory GBM. Similarly, clinical trial NCT03726515 is exploring the combination of EGFRvIII CAR T cells with pembrolizumab, an anti-PD-1 mAb.

### Combination With Oncolytic Viruses

Oncolytic viruses can stimulate immunogenic cell death of cancer cells, production of a type I IFN response in the TME and consequently induction of systemic innate and tumor-specific adaptive immune responses that can promote T-cell trafficking and effector function (Kaufman et al., 2015). Type I IFNs induce clonal expansion, differentiation, development of the cytolytic function, and production of IFN-gamma by CD8 T cells (Curtsinger et al., 2005). In addition, local IFNβ has been shown to disrupt tumor microvasculature (Spaapen et al., 2014), inhibit Treg activation and proliferation (Srivastava et al., 2014), and promote the activity of DCs in TME (Diamond et al., 2011). Therefore, this modulation of the TME is expected to enhance CAR T cell activity (Ajina and Maher, 2017), Huang et al., generated an IL-7-loaded oncolytic adenovirus (oAD-IL7) and used it in combination with B7-H3-specific CAR T cells for the treatment of mice orthotopically grafted with GBM cells (Huang et al., 2021). They demonstrated that the combination of oAD-IL7 and CAR T cells resulted in enhanced T cell proliferation and reduced T cell apoptosis *in vitro*, and prolonged survival, and reduced tumor burden *in vivo*.

### Combination With Small Molecule Inhibitors

There are many pharmacological agents already in clinical use for various diseases which are known to modulate the TME or have effects on GBM cells. As cancer therapeutics, small-molecule inhibitors are designed to block signaling pathways to inhibit tumor growth, survival, angiogenesis, and metastasis. Tyrosine kinase inhibitors (TKIs) have previously been assessed as monotherapies in clinical trials with GBM patients, however, with limited clinical effect (Kim and Ko, 2020). Combination of TKIs with CAR T cells have shown synergistic effects in the treatment of other types of solid cancer (Wu et al., 2019; Huizhong et al., 2020) and therefore are a viable candidate for assessment in GBM. LB-100 is a small molecule inhibitor of protein phosphatase 2A (PP2A), a molecule involved in cell-cell adhesion. Combination with LB-100 can enhance CAIX-specific CAR T cell treatment efficacy both *in vitro* and in *in vivo* models of GBM (Cui et al., 2020).

## Conclusion

CAR T cells are a highly promising therapy for GBM with many potential target TAs identified. Selection of a suitable TA, especially one which is expressed on and will lead to the elimination of GBM CICs, besides that of differentiated GBM cells, but that will allow preservation of normal brain tissue, represents one of the current major challenges in the field. The ability of TA-specific CAR T cells to recognize and eliminate differentiated GBM cells and GBM CICs in preclinical models has been well established. These encouraging results led to the implementation of several clinical trials in patients with advanced GBM (Tables 2, 3).

However, there is still a long way ahead for CAR T cell therapy before it becomes a standard of care for the treatment of patients with GBM. Most clinical trials have proven that CAR T cells as a monotherapy are not particularly effective in solid tumors due to numerous immune escape mechanisms utilized by cancer cells (Lim and June, 2017). So far this appears to also be true for GBM, which additionally presents its own unique challenges to overcome. Among them, optimization of CAR T cell delivery into the brain is an important obstacle to overcome.

Many of the described immune escape mechanisms are not restricted to CAR T cell-based therapy but appear to have a negative impact on many types of immunotherapy. These require the development of combinational strategies to improve the efficacy of immunotherapies and in addition to stimulate the endogenous immune system. As we have described there are many potentially targetable GBM TAs and many CAR-based therapeutic strategies, but it is difficult to decide which strategies are likely to be the most effective. Therefore, the field would benefit from studies which compare the efficacy and associated side effects of each described strategy to determine which one(s) is (are) most likely to translate into significant clinical benefit. Generation of more relevant *in vitro* and *in vivo* models of GBM will help to accelerate and optimize development of CAR T cell treatments (Jacob et al., 2020; Maggs and Ferrone, 2020). For example, patient derived GBM organoids provide a far greater replication of an *in situ* tumor and have been used to model EGFRvIII-specific CAR T cell effects (Jacob et al., 2020). Therefore, whilst there is great promise in the use of CAR T cells to treat GBM, further investigation is needed to optimize the efficacy of this novel therapeutic strategy.

## Author Contributions

LM designed the review, collected the data, analyzed and interpreted the data, and wrote and finalized the manuscript. GC and AD collected the data, analysed and interpreted the data, and wrote the manuscript. ASM collected the data, analysed and interpreted the data, and prepared Figure 1. SF designed the review and wrote and finalized the manuscript. All authors contributed to the article and approved the submitted version.

## Conflict of Interest

The authors declare that the research was conducted in the absence of any commercial or financial relationships that could be construed as a potential conflict of interest.
